# Maternal complication prevention: evidence from a case-control study in southwest Nigeria

**DOI:** 10.4102/phcfm.v6i1.656

**Published:** 2014-12-12

**Authors:** Kayode O. Osungbade, Olubunmi O. Ayinde

**Affiliations:** 1 Faculty of Public Health, College of Medicine, University of Ibadan, Ibadan, Nigeria; 2 University College Hospital, Ibadan, Nigeria; 3 Oyo State Ministry of Health, Ibadan, Nigeria

## Abstract

**Background:**

The importance of strengthening maternal health services as a preventive intervention for morbidities and complications during pregnancy and delivery in developing countries cannot be over-emphasised, since use of prenatal health services improves maternal health outcomes.

**Aim:**

This study investigated differences in risk factors for maternal complications in booked and unbooked pregnant women in Nigeria, and provided evidence for their prevention.

**Setting:**

The study was carried out in a postnatal ward in a secondary health facility.

**Methods:**

This was a case-control study involving booked and unbooked pregnant women who had delivered. Consecutive enrolment of all unbooked pregnant women (cases) was done, and one booked pregnant woman (control) was enrolled and matched for age with each of these. Both groups were interviewed using a questionnaire, whilst records of delivery were extracted from the hospital files. Findings were subjected to logistical regression at a significance level of *p* < 0.05.

**Results:**

Booked women had a lower median length of labour (10 hours) compared to unbooked women (13 hours). More women in the booked control group (139; 35.1%) than in the unbooked case group (96; 23.6%) reported at least one type of morbidity during the index pregnancy (*p* = 0.0004). Booking status was associated with a likelihood of spontaneous vaginal delivery. Young maternal age, low education, rural residence and low socio-economic status were associated with less likelihood of using prenatal services. Young maternal age, low education and intervention in the delivery were associated with a likelihood of experiencing a complication of delivery.

**Conclusion:**

Strengthening antenatal and secondary healthcare services as short- and medium-term measures might be cost-effective as a preventive strategy in complications of pregnancy, whilst socio-economic dimensions of health are accorded priority in the long term.

## Abstrait

**Prévention des complications maternelles au sud-ouest du Nigeria**.

**Historique:**

L'utilisation des services de santé prénatals améliore la santé maternelle.

**But:**

Cette étude examine les facteurs de risques de complications maternelles chez les femmes enceintes prises en charge et celles qui ne le sont pas au Nigeria et apporte des preuves pour prévenir ces complications.

**Cadre:**

un service maternité dans un établissement de santé secondaire.

**Méthodes:**

Une étude de contrôle des cas impliquant des femmes enceintes prises en charge et non prises en charge qui avaient accouché. L'inscription de tous les (cas de) grossesses non pris en charge ont eu lieu consécutivement tandis qu'une femme enceinte prise en charge (contrôle) a été inscrite et appariée selon l’âge avec chacun des (cas) de grossesse non pris en charge. Les deux groupes de participantes ont été interviewés à l'aide d'un questionnaire tandis que des dossiers des accouchements ont été obtenus à partir de la carte de renseignements personnels de chaque participante. Les résultats ont été soumis à la régression logistique à un haut niveau de *P* < 0.05.

**Résultats:**

Les femmes prises en charge avaient aient un accouchement plus court (10 heures) comparé aux 13 heures des femmes non prises en charges. Plus de femmes dans le groupe de ‘contrôle’ pris en charge que dans le groupe de ‘cas’ non pris en charge ont signalé au moins un type de morbidité pendant l'indice de grossesse (139 [35.1%] contre 96 [23.6%]; *P* = 0.0004). La prise en charge allait de pair avec la possibilité d'un accouchement vaginal spontané. Le jeune âge de la mère, le bas niveau d’éducation, le milieu de résidence rural et le faible état socio-économique allaient de pair avec une probabilité moindre d'utilisation des services prénatals. Le jeune âge de la mère, le bas niveau d’éducation et l'accouchement avec intervention extérieure allaient de pair avec la probabilité de complications à l'accouchement.

**Conclusion:**

Le renforcement des services de santé anténatals et secondaires comme mesure à court-et moyen-terme pourrait réduire les prix en tant que stratégie préventive tandis que les dimensions socio-économiques de la santé pourront avoir la priorité à long-terme.

## Introduction

Although recent evidence has shown that not all components of antenatal care are useful in improving maternal and perinatal outcomes,^1, 2^ the role of antenatal care utilisation in achievement of the Millennium Development Goals cannot be under-estimated. Hence the introduction of the concept of focused antenatal care by the World Health Organisation (WHO) and a recommendation of at least four antenatal visits during pregnancy.^3, 4^ However, antenatal care utilisation through a trained health worker has remained far from optimal in developing countries, both in terms of rate of utilisation and quality of services provided. In Nigeria, for example, the antenatal care utilisation rate (i.e. antenatal care clinic attendance at least once during most recent pregnancy) is estimated at only 58% – 60%,^5, 6^ and the rate is much lower when using the WHO standard of antenatal care utilisation. A previous study reported at least four antenatal care visits in 56.6% of women in northern Nigeria during their last pregnancy.^7^

Furthermore, previous studies consistently reported poor quality of maternal health services.^8, 9, 10^ Poor quality of care is one of the most common reasons why women do not choose to use available maternal health services. One study found that perceived quality of service was the most important factor influencing choice of facility for obstetric care.^11^ Similarly, perceived lack of quality in antenatal care was associated with a late first antenatal visit in Kenya.^8^ Therefore low or non-utilisation and declining quality of maternal health services are interwoven challenges which are working against efforts aimed at improving maternal health outcome in developing countries. Apart from quality of care other factors are responsible for low utilisation of the maternal health service, amongst which age, education and socio-economic status are prominent. Young maternal age, low education and low socio-economic status have been associated with lower likelihood of using maternity health services.^6, 8^

Evidence abounds on the relationship between the use of prenatal care services and improved maternal and birth outcomes,^12, 13, 14^ with the maternal mortality rate for unbooked pregnant women reported at 24/1000 and for booked women at 1/1000 in a study in northern Nigeria.^12^ Other researchers reported that unbooked women were 13 times more likely to die in the hospital than booked women.^13^ Likewise, unbooked mothers were twice as likely to deliver preterm babies and three times more likely to have babies with birth asphyxia than booked mothers,^14^ whilst perinatal mortality occurs three times less in booked mothers than in unbooked mothers.^12^ Furthermore, researchers have reported that maternal deaths could be reduced by promoting the availability of, access to and utilisation of basic and comprehensive emergency obstetric care services for women with complications of pregnancy and childbirth.^15^ Effective implementation of such services resulted in a reduction in case fatality rate for direct maternal deaths from 7.2% to 4.6%.^15^ Similarly, adequate use of prenatal care has been linked to higher birthweight and reduced neonatal tetanus; unbooked mothers have been shown to be three times more likely to have low birthweight babies than booked mothers, whilst two or more tetanus immunisations received during prenatal care were protective (adjusted odds ratio [AOR]0.78) against neonatal mortality in sub-Saharan African countries.^16–18^

Despite the potential benefits of maternal health services, pregnant women in developing countries do not utilise these services maximally. This constitutes a barrier to ameliorating maternal morbidity and mortality in such settings. Furthermore, efforts aimed at addressing this challenge are thwarted by lack of commitment of national governments to improve quality of service and also address socio-economic dimensions of health. In view of the foregoing, the importance of strengthening maternal health services as a preventive intervention for dealing with morbidities and complications during pregnancy and delivery in developing countries cannot be over-emphasised. This study therefore investigated differences in risk factors for maternal complications between booked and unbooked pregnant women in Nigeria, and the findings provided evidence on measures for preventing maternal complications and consequently reducing the unacceptably high maternal mortality in developing countries.

## Research methods and design

We carried out a case-control study amongst women who delivered in a secondary healthcare facility over a period of six months.

### Setting

The secondary healthcare facility was the first in Ibadan metropolis, south-west of Nigeria. This area has about 1 million people, and the facility takes referrals from surrounding public and private health facilities. It has a bed capacity of 247 and a health staff comprising 60 physicians (including specialists and medical officers) and 247 nurses. The hospital's records showed that about 410 deliveries occur per month and of these 75 (18.3%) of the women would present with no record of an antenatal visit.

### Study population and sampling strategy

Our study population consisted of all booked (controls) and unbooked (cases) pregnant women who had a delivery (either live or stillbirth) in the health facility within the six months preceding the study. A pregnant woman was categorised as booked (control) if she had made at least two antenatal visits (the second less than two weeks before delivery) or was referred to the hospital with a record of antenatal care. On the other hand, a pregnant woman was categorised as unbooked (case) if she had not made any antenatal visits before delivery or made one or two visits (the second visit more than two weeks before date of delivery), or was referred to the hospital as an emergency for delivery and without any antenatal records at presentation.^19^

Using a prevalence of 58% as the proportion of pregnant women who would attend the antenatal service at least once during their current pregnancy,^5^ 377 booked and 377 unbooked pregnant women were required for the study. All participants were recruited and interviewed in the lying-in ward of the health facility immediately as they arrived and not more two days after delivery, before discharge. All unbooked women who delivered in the hospital during the six-month period were consecutively enrolled. For the purpose of comparison each unbooked woman was matched for age with one booked woman, who was systematically enrolled using a sampling fraction of one-fifth. Written consent was obtained from each participant after providing them with detailed information about the study.

### Data collection

This was carried out using a questionnaire which was developed and pretested in the lying-in ward of a similar health facility. The questionnaire had two parts: the first was used to document sociodemographic characteristics such as marital status, age, parity, socio-economic status, level of education and booking status, and was interviewer-administered in the local language of Yoruba. The second part was used to document delivery records extracted from health facility case notes for each participant. Extracted information comprised obstetric history, age of pregnancy at delivery, duration of labour in hours, method of delivery, complications (during and after delivery) and outcome of pregnancy. A *‘*bidding game approach’ was used to obtain estimates of weekly income.^20^ Each participant was asked about her weekly income by providing a yes/no answer to a suggested amount of money in the local currency. If the answer was ‘no’ to a first suggested amount, which was arbitrarily fixed at 6500 Naira (≈$45) per week, the interviewer made enquiries from the participant as to whether it was an under-estimation or over-estimation in order to determine the direction of bidding. The interviewer then either increased or decreased the bid by increments or decrements of 5000 Naira (≈$35) respectively until the participant said yes. The last sum of money receiving a ‘yes’ response was used as the estimated weekly income for that participant. This was found useful, as it is always difficult for respondents in an informal sector to provide accurate information on weekly income.

### Data analysis

The Statistical Package for the Social Sciences (SPSS 12) was used for data analysis. Mean and standard deviations were estimated for continuous variables and number and percentage were estimated for categorical variables. Students’ t-test was used to compare continuous variables and the chi-square test for categorical variables. The comparison of sociodemographic characteristics, maternal morbidity during pregnancy, mode of delivery and complications of delivery between cases and controls was tested using the chi- square test. Two sets of logistical regression analysis were done, one examining the effect of sociodemographic characteristics (as the independent variables) on booking status (as the dependent variable) and other models examining the effect of booking status (as the independent variable) on outcomes such as mode of delivery, maternal morbidity during pregnancy and complications of delivery (as dependent), with adjustments made for differences in sociodemographic characteristics between cases and controls. For the effect of sociodemographic characteristics on booking status, univariable logistical regression was done, followed by multiple logistical regression analysis including those variables significant at 10% on univariable logistical regression. The unadjusted (univariable) and adjusted (multivariable) odds ratios are presented. The odds ratios comparing unbooked with booked women concerning the outcomes (mode of delivery, maternal morbidity and complications at delivery) after adjusting for differences in sociodemographic characteristics are also stated. Level of significance for all tests was 5%.

### Ethical considerations

Prior to data collection the researchers sought and received approval from the Ethical Research Committee of the State Ministry of Health (AD13/262/114). We also received permission from the authorities of the hospital in which the study was conducted.

## Results

Records showed that 2610 pregnant women delivered in the hospital during the period of the study. Of these, 406 (15.5%) presented in labour as unbooked pregnancies (cases) and were delivered of their babies. Another 406 who also presented in labour, but as booked women, and delivered were enrolled as the comparison group (control). Ten of the booked women were either discharged or referred before they could be interviewed.

The ages of all the studied women were between 16 and 44 years, with a mean of 26.6 ± 5 years. The mean age of booked women was 26.9 ± 4 years compared to 26.4 ± 5.2 years for unbooked women (*p* = 0.12). Most of the studied women, 724 (90.2%), were aged 20 to 35 years; others were either less than 19 or more than 36 years old (Table 1). More of the unbooked pregnant women (307 or 75.6%) were semi-urban and rural residents, with 276 (69.7%) booked women of similar residence (*p* = 0.0326). Seventy-two (18.2%) of the booked women were single mothers compared to 116 (28.6%) of the unbooked women (*p* = 0.0008). More than half of the women (484 or 60.3%) had a secondary education, 189 (23.6%) had primary and 84 (10.5%) a tertiary education. A significantly lower proportion of booked women (15 or 3.8%) had no formal education compared to 30 (7.4%) of unbooked women. Concerning tertiary education, a significantly higher proportion of booked women (56 or 14.1%) had a tertiary education compared to 28 (6.9%) of unbooked women (*p* = 0.0009) (Table 1). The parity of all the studied women was between 0 and 6 children, with a mean of 2.3 ± 1.4 children. The mean parity of booked women was 2.3 ± 1.4 children compared to 2.3 ± 1.5 children for unbooked women (*p* = 0.5). Almost all of the women, 775 (96.6%), self-reported a lower weekly income category of N6500 (≈$45) per week (Table 1).

**TABLE 1:** Characteristics of the studied women.

Characteristics	Sub-category	Booking status				Total		*p* value
	
		Booked (*n* = 396)		Unbooked (*n* = 406)		*n*	%	
	
		*n*	%	*n*	%			
Age group (yrs)	≤ 19	15	3.8	24	5.9	39	4.9	0.3264
	20–29	261	65.9	269	66.3	530	66.0	
	30–35	103	26.0	91	22.4	194	24.2	
	≥ 36	17	4.3	22	5.4	39	4.9	
Residence	Urban	120	30.3	99	24.4	219	27.3	0.0326
	Semi-urban	269	68.0	290	71.4	559	69.7	
	Rural	7	1.7	17	4.2	24	3.0	
Marital status	Single	72	18.2	116	28.6	188	23.4	0.0008
	Married	317	80.0	288	70.9	605	75.4	
	Divorced	7	1.8	2	0.5	9	1.2	
Educational Status*	No education	15	3.8	30	7.4	45	5.6	0.0009
	Primary	84	21.2	105	25.9	189	23.6	
	Secondary	241	60.8	243	59.8	484	60.3	
	Tertiary	56	14.1	28	6.9	84	10.5	
Weekly Income (1 US $ ≈ N143)	≤N6500	376	94.9	399	98.3	775	96.6	0.009
	>N6500	20	5.1	7	1.7	27	3.4	
Disease	Any antenatal morbidity	**139**	**35.1**	**96**	**23.6**	**235**	**29.3**	**0.0004**
	Malaria	93	23.5	47	11.6	140	17.5
	Hypertension	10	2.5	7	1.7	17	2.1
	Antepartum haemorrhage	6	1.5	18	4.4	24	3.0
	Infections?	17	4.3	7	4.4	24	3.0
	Premature rupture of membranes	13	3.3	17	4.2	30	3.7
Methods of delivery	Spontaneous vaginal delivery	364	91.9	348	85.7	712	88.8	0.0176
	Caesarean section	23	5.8	45	11.1	68	8.5	
	Assisted vaginal delivery‡	9	2.3	13	3.2	22	2.7	
Complications at delivery	Any delivery complication	**92**	**23.2**	**93**	**22.9**	**185**	**23.1**	**0.9128**
	Perineal tears	59	14.9	16	3.9	75	9.4	
	Retained placenta	7	1.8	31	7.6	38	4.7	
	Intrapartum haemorrhage	12	3.0	21	5.2	33	4.1	
	Eclampsia	8	2.0	10	2.5	18	2.2	
	Foot drop§	5	1.3	9	2.2	14	1.7	
	Malaria	2	0.5	7	1.7	9	1.1	
	Sepsis	3	0.8	9	2.2	12	1.5	
	Anaemia	5	1.3	18	4.4	23	2.9	

* Educational status – highest educational level attained.

† Infections – urinary tract infection, respiratory tract infection, vaginal candidiasis.

‡ Assisted vaginal deliveries – forceps, vacuum, assisted breech.

§ Foot drop – this is usually a temporary condition caused by compression of the nerve that controls the muscles involved in lifting the foot especially following a difficult labour. It causes weakness or paralysis of the muscles and makes it difficult or impossible for women to walk after child delivery.

Overall the number of hours in labour ranged from 3 to 23 hours, with a median of 12 hours. Booked women had a lower median of hours of labour (10 hours) compared to 13 hours recorded for unbooked women. More women in the booked ‘control’ group than in the unbooked ‘case’ group reported at least one type of morbidity during the index pregnancy (139 [35.1%] versus 96 [(23.6%]; *p* = 0.0004). Reported morbidities during pregnancy included malaria, hypertension, antepartum haemorrhage, other infections (bacterial or fungal) and premature rupture of the membranes (Table 1). Most of the women (712 or 88.8%) had a spontaneous vaginal delivery, whilst 90 (11.2%) had an intervention delivery. Of the 712 women who had a spontaneous vaginal delivery, 364 (91.9%) were booked and 348 (85.7%) were unbooked women; likewise, of the 68 women who had a caesarean section, 23 (5.8%) were booked compared to 45 (11.1%) women who were unbooked; these differences were statistically significant (*p* = 0.0176). Similarly, of the 22 women who had an assisted vaginal delivery, 9 (2.3%) were booked compared to 13 (3.2%) who were unbooked; however, this difference was not statistically significant (*p* = 0.42).

During delivery 209 women were given an episiotomy; of these, 137 (34.6%) were booked and 72 (17.7%) were unbooked. About equal proportions of women in the two groups (92[23.2%] booked versus 93 [22.9%] unbooked; *p* = 0.9128) reported at least one type of complication during delivery of the index pregnancy. These complications included perineal tears, retained placenta, intrapartum haemorrhage, eclampsia, foot drop, sepsis and anaemia (Table 1). One maternal death resulting from prolonged obstructed labour was reported amongst the unbooked women.

Pregnant women who were rural residents were half as likely to book for antenatal care as those who resided in urban and semi-urban areas (AOR = 0.45; 95% confidence interval [CI] 0.17–1.18) (Table 2). Pregnant women in the reproductive age group were more likely to use prenatal services than adolescent or elderly gravida women (OR for those ≤ 19 years old 0.64 [95% CI 0.33–1.26] and 0.80[0.41–1.53] for those aged ≥ 36 years). Single pregnant women were less likely to use prenatal services than married or divorced women (AOR = 0.58; 95% CI 0.41–0.81). Women of low education were less likely to use prenatal services than women with at least secondary education (AOR for women with no formal education 0.46 (95% CI 0.24–0.90) and for women with primary education 0.82 (0.58–1.15)). Women with a higher weekly income of 6500 Naira (≈$45) and above were twice as likely to use prenatal services than those who earned less (AOR = 2.07; 95% CI 0.84–5.13).

**TABLE 2:** Univariate and multivariate analyses of factors associated with booking for antenatal care by women.

Variable category	Sub-category	Booking for antenatal care	

		Univariate logistical regression, unadjusted OR (95% CI)	Multivariable logistical regression, adjusted OR (95% CI)
Residence	Urban	1.00	1.00
	Semi-urban	1.31 (0.96–1.79)	1.36 (0.98–1.87)
	Rural	0.50 (0.19–1.25)	0.45 (0.17–1.18)
Age group (yrs)	20–29	1.00	
	≤ 19	0.64 (0.33–1.26)	
	30–35	1.17 (0.84–1.62)	
	≥ 36	0.80 (0.41–1.53)	
Marital status	Married	1.00	1.00
	Single	0.56 (0.40–0.79)	0.58 (0.41–0.81)
	Divorced	6.36 (0.78–52.0)	4.83 (0.94–24.6)
Educational status	Secondary education	1.00	1.00
	No formal education	0.52 (0.27–0.99)	0.46 (0.24–0.90)
	Primary education	0.81 (0.58–1.13)	0.82 (0.58–1.15)
	Tertiary education	2.01 (1.24–3.28)	1.64 (0.99–2.74)
Minimum wage	≤ N6500	1.00	1.00
	> N6500	3.03 (1.27–7.25)	2.07 (0.84–5.13)

Table 3 shows that pregnant women who used prenatal services were about one and a half times more likely to experience at least one morbidity during pregnancy than women who did not use them (AOR = 1.69; CI 1.23–2.32). Women of low education were less likely to experience a morbidity during pregnancy than women with at least secondary education (AOR for women with no formal education 0.89 [95% CI 0.42–1.88] and 0.90 [0.61-1.34] for women with primary education). There were no significant differences between booked and unbooked groups in terms of tendency to experience complications during delivery (Table 1), although certain sociodemographic characteristics were found to influence this tendency. Adolescent pregnant women were more likely to experience a complication during pregnancy than older women (AOR = 1.13; CI 0.52–2.42). Women who gave birth by assisted vaginal delivery (AOR 1.37; 95% CI 0.51–3.69) and caesarean section (AOR 5.96; 3.45–10.29) were about one and half and six times more likely to have experienced a complication during pregnancy respectively. Higher weekly income earners were three times more likely to experience a complication during pregnancy than women in the low weekly income group (AOR = 2.94; CI 1.20–7.20). Women of low education (AOR 1.46; 95% CI 0.62–3.41) were about one and half times more likely to experience a complication during pregnancy than women with at least secondary education (AOR 1.61; CI 1.06–2.44).

**TABLE 3a:** Univariate and multivariate analyses of factors associated with experiencing any morbidity during pregnancy and complications during delivery.

Characteristics	Sub-category	Morbidity during pregnancy	

		Univariate logistical regression, unadjusted OR (95% CI)	Multivariable logistical regression adjusted OR (95% CI)
Booking status	Unbooked	1.00	1.00
	Booked	1.75 (1.28–2.38)	1.69 (1.23–2.32)
Educational status	Secondary education	1.00	1.00
	No formal education	0.81 (0.39–1.64)	0.89 (0.42–1.88)
	Primary education	0.85 (0.58–1.24)	0.90 (0.61–1.34)
	Tertiary education	1.82 (1.13–2.93)	1.85 (1.11–3.06)

**TABLE 3b:** Univariate and multivariate analyses of factors associated with experiencing any morbidity during pregnancy and complications during delivery.

Characteristics	Sub-category	Complications during delivery	

		Univariate logistical regression, unadjusted OR (95% CI)	Multivariable logistical regression adjusted OR (95% CI)
Age group (yrs)	20–29	1.00	1.00
	≤ 19	1.72 (0.87–3.41)	1.13 (0.52–2.42)
	30–35	0.58 (0.38–0.90)	0.57 (0.35–0.93)
	≥ 36	1.06 (0.50–2.23)	0.96 (0.42–2.18)
Mode of delivery	Spontaneous vaginal	1.00	1.00
	Assisted vaginal	1.49 (0.57–3.88)	1.37 (0.51–3.69)
	Caesarean section	4.47 (2.68–7.45)	5.96 (3.45–10.29)
Income group	≤ N6500	1.00	1.00
	> N6500	1.70 (0.75–3.85)	2.94 (1.20–7.20)
Educational status	Secondary education	1.00	1.00
	No formal education	0.87 (0.40–1.87)
	Primary education	1.25 (0.85–1.84)	1.61 (1.06–2.44)
	Tertiary education	0.73 (0.40–1.34)	0.78 (0.40–1.51)

## Discussion

This study reported a prevalence of unbooked pregnancies of 15.5%. This is evidence of low or non-utilisation of antenatal care as a persistent and common practice in the study setting, confirming previous reports of 17% – 29% from similar surveys.^13, 14^ This study reported a significantly higher occurrence of maternal morbidity amongst the booked control group than the unbooked cases. This finding could be considered to be one of the benefits of antenatal care received by booked women, as it might have made possible early detection and prompt management of the reported morbidities during pregnancy. Booked women would also have had their delivery plans discussed and instituted with healthcare workers. These interventions, coupled with skilled delivery care received and the higher socio-economic parameters of booked women, might have been responsible for the improved maternal and perinatal outcomes amongst the study participants. The low reporting of morbidities by unbooked women might have partly resulted from under-reporting and missed diagnosis of the conditions; for example, the reported considerable use and abuse of antimalaria drugs and antibiotics, which are easily purchased across the counter for self-treatment in the study setting,^21^ might be responsible for the low rate of malaria and infections of the urinary, respiratory and vaginal tracts.

As observed above, low or non-utilisation of antenatal care is a common practice in the study setting; in addition, out-of-hospital delivery is high, as 62.1% of deliveries reportedly occurred at home.^5^ These factors, amongst others, may contribute to the different experiences reported amongst the two groups of women with regard to normal and intervention deliveries. Caesarean section and assisted delivery occurred more frequently amongst the unbooked women than their booked counterparts. This finding could be viewed as a direct output of the skilled delivery care received by the unbooked women, as the procedures might have been interventions aimed at dealing with the above-reported maternal complications, thereby saving the lives of mother and child.

From the foregoing, the common occurrence of unbooked emergencies with their attendant complications and potential contribution to maternal mortality seem to justify the proposition that promoting the availability of, access to and utilisation of basic and comprehensive emergency obstetric care services for women with complications of pregnancy and childbirth should be emphasised more than routine antenatal care services as a means of reducing maternal mortality in developing countries.^15^ Although the cost of this ‘preventive’ mitigating strategy could be enormous in a resource-poor setting, the number of lives of women expected to be saved would evidently outweigh the cost consideration, particularly as emergencies are highly prevalent in unbooked women.

In view of the above it is suggested that health planners and care providers should accord high priority to strengthening the health systems with capacity to provide prompt and effective interventions against pregnancy and labour complications, particularly for unbooked pregnancies. Consequently health facilities should be appropriately staffed with personnel who are capable of providing the desired services. Equipping healthcare facilities to provide basic and comprehensive emergency obstetric care services sufficient to deal with such complications in developing countries requires specific training for health workers and provision of state of the art equipment and facilities for detecting, resuscitating and managing life-threatening situations. The capacity of midwifery and nursing personnel could be enhanced through WHO training on life-saving skills;^22^ likewise, medical personnel require skill building in cardiopulmonary resuscitation as well as assisted and operative delivery to be able to intervene when necessary. Staff training should be supported by provision and maintenance of appropriate equipment and facilities optimally required to provide the desired services, including blood transfusion services, in the health facilities.

In order to ensure efficiency in the health systems, the policy on human resources for health should address strategies (such as staff redeployment and retention) which are required to make available healthcare staff in a manner to favour rural residents in particular.^23^ Furthermore, best practices dictate a continuous review of health systems, as such an exercise provides the opportunity for appraisal and identification of strengths and weaknesses requiring specific interventions. It is highly desirable to subject the above recommended measures to a periodical clinical audit for maintenance of quality care at all times.

Whilst the above recommendations could suffice as short- and medium-term measures, we further suggest that healthcare planners and providers institute long-term cost-effective measures aimed at addressing the high maternal mortality. It has previously been shown that antenatal care could positively influence a key strategy of maternal mortality reduction in developing countries – that is that all pregnant women deliver with the assistance of a skilled attendant.^24^ Unfortunately the situation with regard to skilled delivery assistance in the study setting is poor; this was recently reported to be 39% – 44%.^5, 6^ Therefore strategies aimed at increasing the current antenatal care utilisation rate of 58% – 60% should be vigorously pursued alongside improving the quality of service. It is hoped that increasing the utilisation rate of antenatal care services would have a direct impact on use of delivery services manned by skilled health workers.

National governments and international development partners might place priority on other long-term strategies such as education of every girl child and improving household wealth in poor-resource settings, especially for female populations. It is expected that these measures will have multidimensional effects. Firstly, girls’ education would help them to develop essential life skills, including self- confidence, ability to participate effectively in society, and to protect themselves from harmful sociocultural practices, including late or non-booking for antenatal care. Secondly, educational attainment of girls and improvement in household wealth would empower women economically and in decision making to seek health care. Thirdly, female education might help to keep women in school until they are of an age which would favourably use maternal health services.

## Conclusion

Unbooked pregnant women presenting in labour is a common phenomenon amongst the study population, and might be contributing significantly to the high maternal mortality rates in developing countries. Whilst efforts at provision of basic and comprehensive emergency obstetric care services for such women are currently being emphasised as a means of reducing maternal mortality, strategies aimed at addressing the low or non-utilisation of maternal health services are equally important, and hence require adequate attention.
